# LiverColor: An Artificial Intelligence Platform for Liver Graft Assessment

**DOI:** 10.3390/diagnostics14151654

**Published:** 2024-07-31

**Authors:** Gemma Piella, Nicolau Farré, Daniel Esono, Miguel Ángel Cordobés, Javier Vázquez-Corral, Itxarone Bilbao, Concepción Gómez-Gavara

**Affiliations:** 1 Engineering Department, Universitat Pompeu Fabra, 08018 Barcelona, Spainmiguelangel.cordobes@upf.edu (M.Á.C.); 2 Computer Vision Center and Computer Sciences Department, Universitat Autònoma de Barcelona, 08193 Barcelona, Spain; jvazquez@cvc.uab.cat; 3 Servicio de Cirugía HBP y Trasplante, Hospital Universitari Vall d’Hebron, Vall d’Hebron Institute of Research (VHIR), 08035 Barcelona, Spainconcepcion.gomez@vhebron.cat (C.G.-G.)

**Keywords:** mobile app, colour and texture analysis, liver assessment, organ transplantation, hepatic steatosis

## Abstract

Hepatic steatosis, characterized by excess fat in the liver, is the main reason for discarding livers intended for transplantation due to its association with increased postoperative complications. The current gold standard for evaluating hepatic steatosis is liver biopsy, which, despite its accuracy, is invasive, costly, slow, and not always feasible during liver procurement. Consequently, surgeons often rely on subjective visual assessments based on the liver’s colour and texture, which are prone to errors and heavily depend on the surgeon’s experience. The aim of this study was to develop and validate a simple, rapid, and accurate method for detecting steatosis in donor livers to improve the decision-making process during liver procurement. We developed LiverColor, a co-designed software platform that integrates image analysis and machine learning to classify a liver graft into valid or non-valid according to its steatosis level. We utilized an in-house dataset of 192 cases to develop and validate the classification models. Colour and texture features were extracted from liver photographs, and graft classification was performed using supervised machine learning techniques (random forests and support vector machine). The performance of the algorithm was compared against biopsy results and surgeons’ classifications. Usability was also assessed in simulated and real clinical settings using the Mobile Health App Usability Questionnaire. The predictive models demonstrated an area under the receiver operating characteristic curve of 0.82, with an accuracy of 85%, significantly surpassing the accuracy of visual inspections by surgeons. Experienced surgeons rated the platform positively, appreciating not only the hepatic steatosis assessment but also the dashboarding functionalities for summarising and displaying procurement-related data. The results indicate that image analysis coupled with machine learning can effectively and safely identify valid livers during procurement. LiverColor has the potential to enhance the accuracy and efficiency of liver assessments, reducing the reliance on subjective visual inspections and improving transplantation outcomes.

## 1. Introduction

High organ quality is key to a successful transplant outcome. Unfortunately, assessing organ quality is a challenging task, and there is currently no practical evaluation method available to help the surgical team decide whether to accept or discard an organ. In the case of the liver, hepatic steatosis (HS) is frequently encountered during procurement surgery [1], and it is the main reason for declining the donor’s liver due to the increased risk of postoperative complications [2,3,4,5,6]. 

Hepatic steatosis is characterised by increased fat accumulation in the liver cells. It is the most prevalent of all liver disorders, affecting approximately 30% of the general population [7]. The disease shows few or no symptoms, which makes it difficult to diagnose until it presents complications. In the context of transplantation, HS in the donor is a serious problem because steatotic livers are vulnerable to preservation damage, resulting in a higher risk of early allograft dysfunction and primary nonfunction [5,8]. Liver biopsy is the gold standard for evaluating HS, but it is invasive (hence, it can damage the organ), costly, slow, and not always available during liver procurement [7]. Moreover, it only samples a small fraction of the organ and, since HS is often unevenly distributed throughout the liver, there is the potential for significant sampling error [9]. For these reasons, in practice, the decision to use or discard the organ is based on the surgeons’ visual assessment (colour and texture) of the liver during procurement [6,10,11]. Fatty livers exhibit some degree of yellowness and a coarser texture, which can be observed macroscopically in the graft without the need for imaging tests. While this visual inspection is fast, it is subjective and highly error-prone, relying heavily on the surgeon’s experience [11,12]. In cases of doubt, clinicians tend to err on the side of caution and discard the liver, despite organ shortages and growing waiting lists. It is estimated that around 50% of the livers discarded based on visual assessment could have been used for transplantation if an accurate, objective evaluation had been performed [10,13]. This situation has prompted an urgent need to develop a reliable, cost-effective, and fast method to support surgeons in their decision-making process regarding the acceptance or rejection of liver grafts, thereby avoiding the unjustified loss of organs. 

Computer tomography, magnetic resonance imaging, ultrasound, and spectroscopy have been explored for assessing HS, albeit with limited success in the context of liver transplantation [11,14,15,16]. Some studies have evaluated the use of photographs to assess steatosis, either from biopsy samples [17] or directly from the liver graft itself [18,19]. Cesaretti et al. [18] developed a texture-based support vector machine (SVM) classification algorithm on a cohort of 56 liver grafts, achieving an accuracy of 89% when combined with donor data. However, the donor characteristics of their transplantable livers significantly differed from the non-transplantable ones, which suggests a potential representation bias in the learned model, and it is unclear whether their algorithm may generalise well. Similarly, Ugail et al. [19] combined pre-trained deep learning networks with various classifiers to discriminate between valid and non-valid livers. They report accuracies of up to 99.6%. However, they split the data into training and test sets at the feature level rather than at the donor level, which suggests a potential data leakage. Nonetheless, these studies show the growing interest in rapidly and accurately assessing liver quality at the point of organ procurement to safely expand the donor pool. 

Our study aimed to present and evaluate LiverColor, a software-based platform that encompasses a new diagnostic method for assessing HS using colour image processing coupled with machine learning on standard photographs of livers.

## 2. Materials and Methods

We used an agile co-design approach to develop LiverColor, involving three key stakeholder groups: the research team (comprising engineers and clinicians), the target user group (including transplant teams and other healthcare professionals), and the software development team. Together, these stakeholders shaped the design of LiverColor, which includes 4 main interrelated components: (1) the database and repository of images and clinical data; (2) the mobile application; (3) the image processing and data analysis tools based on machine learning; and (4) the web portal application. We used extreme programming (XP) as an agile software development methodology [20]. It is based on a set of rules and good practices for software development in highly changing environments, and it is focused on continuous feedback between the development team and the user (the transplant clinicians and nurses in our case). Only open-access software was used for the development of LiverColor. 

### 2.1. Database and Repository

**Figure 1:**
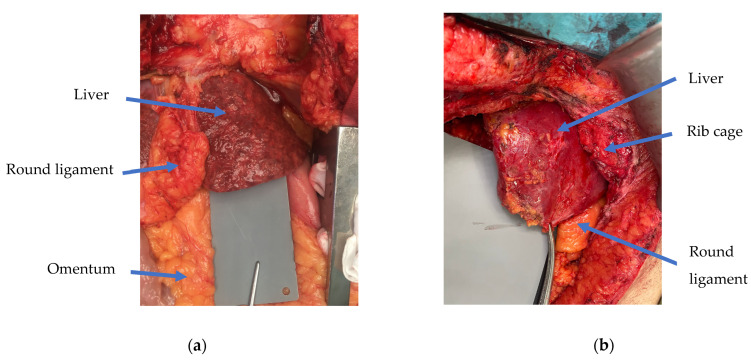
Examples of images in the training dataset: (**a**) liver with hepatic steatosis > 30% and (**b**) liver with hepatic steatosis < 30%, as quantified by biopsy. The grey card that is visible next to the liver ensures colour balance.

Data were collected by clinicians from Vall d’Hebron University Hospital and used by the guidelines set by the hospital’s Ethical Research Committee (CEIC) and the current legislation (Organic Law 15/1999). The in-house dataset consisted of 192 cases from brain-dead donors. All pictures were taken by surgeons in a well-lit operating room with the surgical light switched off, using mobile phones equipped with (at least) a 12-megapixel camera and high-end optics, features commonly available in today’s latest-generation mobile phones. To ensure colour calibration, a sterilised plastic grey card was placed next to the liver. For each liver, up to 5 photographs were taken: two from each lobe (before and after surgical biopsy) and an additional one after cold organ perfusion (i.e., back-table procedure). In total, 362 photographs were obtained. All livers underwent two separate tru-cut needle biopsies, one for each lobe, to determine the degree of steatosis. Figure 1 shows two examples of images from the training dataset, with one depicting a liver with HS > 30% (Figure 1a) and another showing a case with HS < 30% (Figure 1b). Demographic information about the subjects (e.g., sex, age, and body mass index) and biochemical variables, -aspartate aminotransferase (AST), alanine transaminase (ALT), gamma-glutamyl transferase (GGT), and bilirubin- were also recorded. Table 1 summarises baseline characteristics of the donors. 

All data are managed through the Firebase platform, which provides different services for the development of web and app applications, including the following:

- User authentication;
- Registration, manipulation, and extraction of data (database);
- Configuration of data access permissions;
- Registration, manipulation, and reading of images and files (repository);
- Configuration of access permissions to images and files;
- Website hosting;
- Data encryption.

### 2.2. Mobile Application

The mobile app allows for easy storage and real-time analysis of data and images from donors’ livers. It includes the following functionalities:

- Log in;
- Management of user information;
- Creation of new cases;
- Registration of donor’s data;
- Registration of the liver viability according to the surgeon;
- Registration of biopsy results;
- Image acquisition;
- Image calibration and analysis (see Section 2.3);
- Management of cases (delete, archive, etc.).

All these functionalities have been developed considering the demanding environment in which the app will be used. For the medical team, organ procurement is stressful due to the time pressure and the critical impact of their decisions on the recipient’s life expectancy. Consequently, it is important to build a robust application that (1) is fast and easy to use, minimising any elements that may confuse, loss of data, loss of time, or human error; (2) ensures privacy and data protection. For this, data are encrypted both in their transfer and storage, adding double encryption on the client side to avoid the traceability of the data if third parties gain access to the database.

### 2.3. Image Processing and Data Analysis Tools

The analysis includes image calibration, feature extraction from the liver region, and finally classification using machine learning. Image analysis and classification models were implemented using open-source libraries in Python v3.11.4.

#### 2.3.1. Image Calibration and Feature Extraction

Mobile phones typically represent and display intensities in an sRGB colour space. Thus, to recover the original linear intensity response from the photographs, the gamma encoding is reversed by raising each pixel value to the power of 2.2 [21]. After this step, the image is calibrated to mimic acquisition under uniform white lighting conditions, using the grey card colour as a reference. Subsequently, the liver is segmented from the calibrated image using a fully automatic convolutional neural network based on the nn-Unet architecture [22]. Given that HS may not be homogeneous, we divide the liver into several randomly selected non-overlapping patches rather than analysing the entire organ. The number and size of these patches depend on the area of the liver in the photograph. On average, each liver is divided into 20 patches, with a range of 15 to 25 patches, each measuring 80 × 80 pixels, within a range of 60 × 60 to 120 × 120 pixels. Using patches has the added advantage of accelerating computational time. Patches with specular highlights are identified using luminance thresholding [23]. Specifically, the mean luminance of the patch is compared with the interquartile range of the liver’s luminance values. If the mean luminance of the patch falls outside this range, the patch is excluded from further processing and replaced with another randomly selected patch that meets the criteria. Colour and texture features are computed from the patches. For the colour features, we use the histograms of the L*a*b* intensities. In the L*a*b* colour space, a* and b* are chromaticity axes. The a* indicates the green-red component colour (with negative a* values towards green and positive towards red), whereas the b* represents the blue-yellow component (with negative values towards blue and positive towards yellow). The opponent colour model is well-suited for our application because steatotic livers are less red and have a yellow undertone. For texture features, we use histograms of local binary patterns (in the L*a*b* colour space) due to their ability to describe local texture [24]. 

In addition to the colour and texture features extracted from the image, LiverColor allows for the inclusion of donor data (age, sex, body mass index, AST, ALT, GGT, and bilirubin) to be considered for the prediction.

#### 2.3.2. Training and Validating the Classification Model

LiverColor provides various trained models, with different types of machine learning models (SVM and random forests), different training images (pre-biopsy and after perfusion), and different parameters (HS threshold at 15% and 30% and using only image features or combining these with clinical data). If the biopsy result of the liver exceeds the HS threshold, the ground-truth label assigned to the organ is “non-valid”; otherwise, it is labelled as “valid”. We used nested cross-validation, with an outer cross-validation loop to split the data into training and test folds (70% and 30%, respectively), and an inner loop (90% and 10%) in combination with grid-search to select the optimal hyperparameters. Training and test data partition was conducted at the donor level (rather than at the patch level) to avoid data leakage, and it was random stratified (i.e., the proportion of the different classes in the training dataset was kept constant) to combat covariate shift due to class imbalances in the data. Moreover, cost functions for the classifiers were weighted inversely proportional to the corresponding class frequency to address class imbalance.

During inference, each patch is classified by the trained machine learning model as either valid (HS ≤ threshold%) or non-valid (HS > threshold%). A final classification for each liver is determined based on the proportion of non-valid patches: if a specified percentage of the patches (default value set at 20%) in the organ are classified as non-valid, the entire liver is estimated as non-valid; otherwise, it is considered as valid. 

The classification performance was evaluated by predicting the classes of all livers in the test dataset (using the classifier that was trained on the training dataset) and comparing the predictions against the ground-truth class labels of the test dataset. The average of the accuracies obtained from the outer cross-validation loop was considered to be the generalisation performance. In addition to accuracy, precision, and recall, we evaluated the classification performance by computing the receiver operating characteristic curve (ROC) and its area under the curve (AUC). Results are reported as point estimates and their associated 95% confidence interval (CI). 

We compared LiverColor’s performance against the standard of care. Transplant surgeons, blind to LiverColor predictions, were asked to provide a qualitative assessment of the HS in the test dataset.

### 2.4. Web Portal

From the web portal, the administrator can manage users and monitor their activity, visualise and analyse the cases, register new pre-trained machine learning models, and obtain key performance indicators of the models in use. More specifically, the administrator can:

- Create, edit, and delete users;
- Visualise statistics of the cases registered by user and by centre;
- Download data in csv format;
- Visualise, manage, and filter cases;
- Register new machine learning models;
- View behaviour and performance statistics for each of the models in use.

### 2.5. Data Flow and Backend Architecture

**Figure 2:**
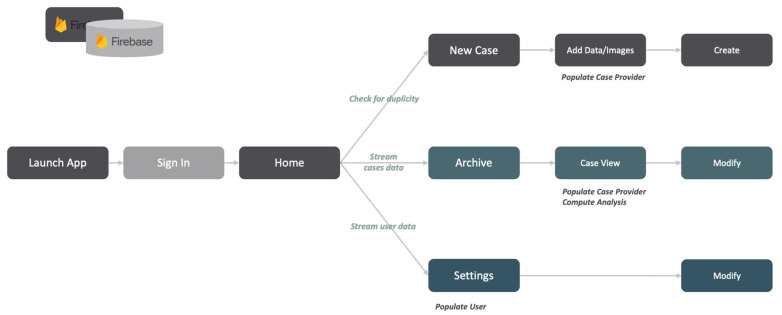
Data flow in LiverColor.

Figure 2 shows the data flow in the application, that is, how data are passed along through the app from the launch to display, and how that is structured.

We used Flutter and Dart for the front-end, and Flask for the backend engine to deploy the machine learning models. Flutter is an open-source development framework for building cross-platform native mobile applications, with Dart as its client-optimised programming language. In contrast, Flask is a lightweight Python backend framework for web applications.

**Figure 3:**
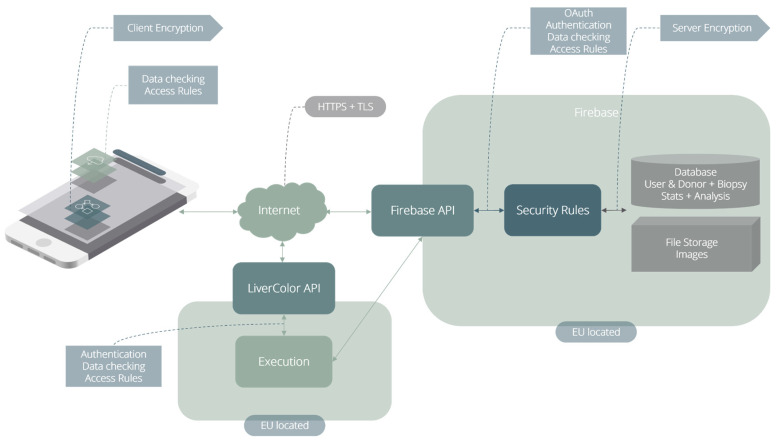
The backend architecture of LiverColor.

Figure 3 depicts the backend architecture. It contains two application programming interfaces (APIs) for communication between the client, the database (DB), and the computation engine. The client can only communicate via HTTPS with the Firebase APIs and the LiverColor API. Firebase Authentication, which is the service for user authentication, encrypts the user data and leverages industry standards such as OAuth 2.0 (for user authorisation and access control to the data) and OpenId Connect (for authentication).

For data storage, LiverColor uses Firestore, the non-relational cloud database of Firebase. Firestore automatically encrypts all data using the 256-bit Advanced Encryption Standard, and the encryption keys themselves are encrypted with a set of regularly rotating master keys. In addition, in the treatment of sensitive data, the encryption system by the server (provided by Firebase) is combined with a client-side encryption and decryption system, to anonymise data and avoid their traceability. Data are transparently decrypted when read by an authorised user. 

### 2.6. LiverColor’s Evaluation

For the platform’s evaluation, test procedures were defined in three steps: (1) testing of each individual module (i.e., access to repository, mobile application, image and data analysis, web portal), (2) system test to verify the integration of each module within the platform, and (3) functional test to assess the overall system performance, in an operational environment, including the assessment of the tools usage. Classification performance was evaluated as explained in Section 2.3.2. To assess the impact of gamma correction and colour calibration (Section 2.3.1) on classification performance, we conducted experiments without applying these corrections under two scenarios: (a) using the original images and (b) using modified images with varied ambient light to simulate different lighting conditions.

We assessed the app’s usability through a thorough, scenario-based summative evaluation of user–platform interactions, employing heuristic analysis [25] and a mixed methods approach [26]. It involved collecting quantitative data to measure specific metrics (e.g., accuracy of the classification and completion times) and qualitative data, including user feedback and opinions. A/B testing was applied to determine the effect of various design optimisations. User experience was evaluated by a panel of experts (nine experienced transplant surgeons) using the mHealth app usability questionnaire (MAUQ) [27]. The questionnaire consists of 18 statements about the ease of use and satisfaction, system information arrangement, and usefulness, and an open “Additional comments” section. Responses to the positive statements range from 1 (strongly disagree) to 7 (strongly agree). To determine the usability of LiverColor, we calculated the average of the responses to all statements. The higher the overall average, the higher the usability of the app.

## 3. Results

**Figure 4:**
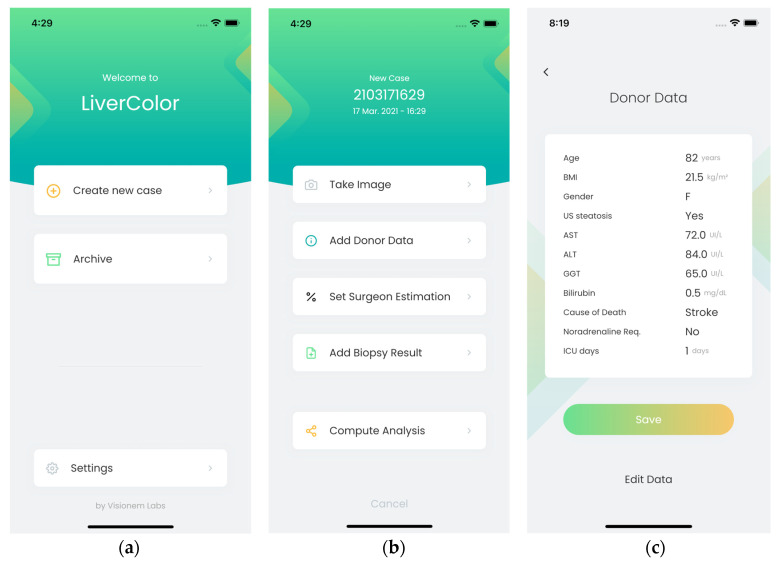
Screenshots of the mobile app: (**a**) after login, users can either create a new case or consult an already existing one; (**b**) when creating a new case, users take a picture of the liver and register the donor’s data. The surgeon’s estimation of steatosis and the results of the biopsy can also be registered; (**c**) donor’s data that can be included.

**Figure 5:**
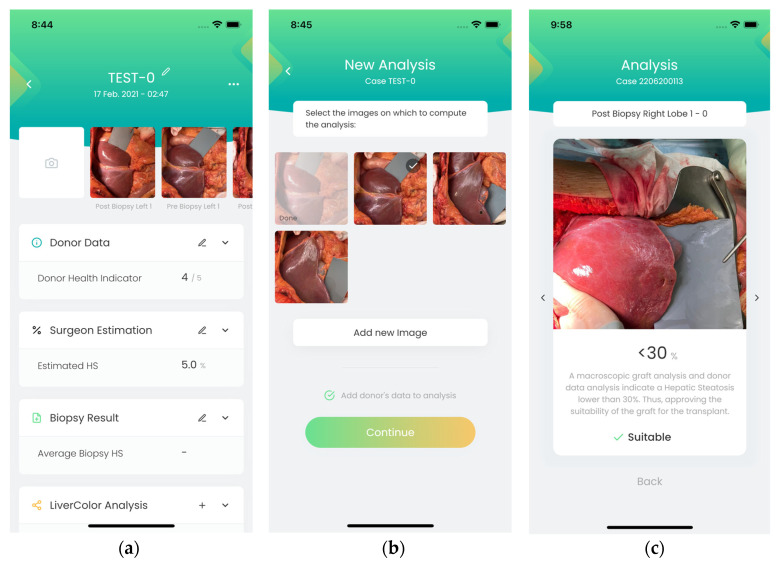
Screenshots of the mobile app: (**a**) edition of some input variables; (**b**) the user can select the image to evaluate and whether donor’s data should be used in the prediction; (**c**) result of HS evaluation.

**Figure 6:**
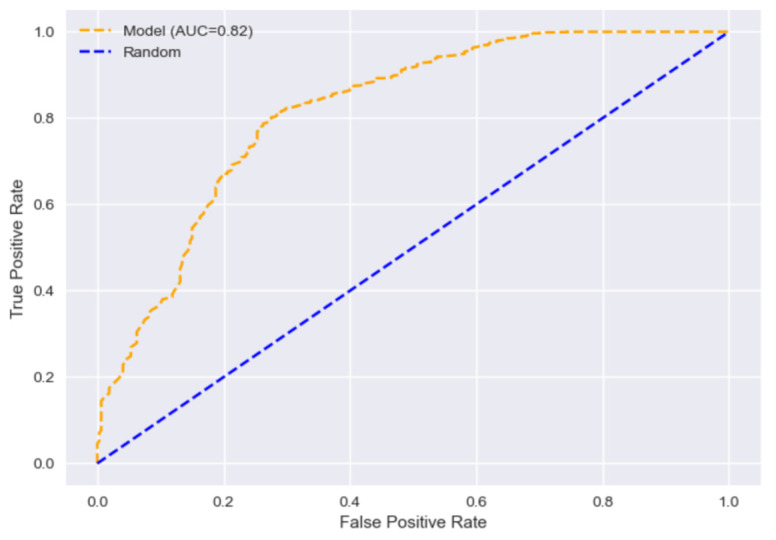
ROC curve using the random forest classifier trained on both image and donor clinical data to predict hepatic steatosis above 15%.

**Figure 7:**
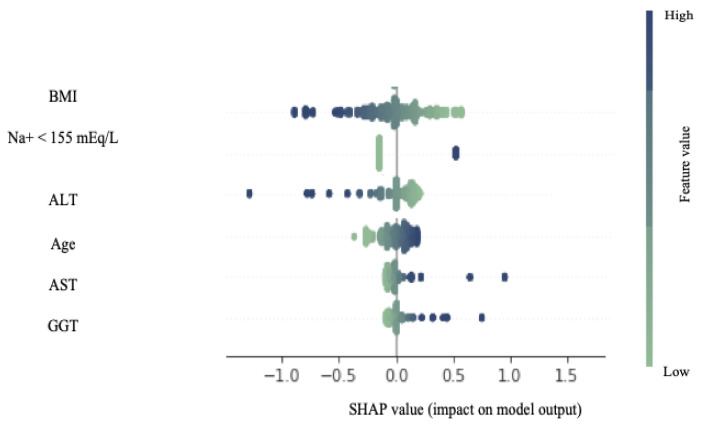
Shapley additive values for the metadata. Each liver case is represented by a single dot on each feature row. The colour indicates the relative attribute values of the feature, with blue indicating high numerical values and green indicating low numerical values. The horizontal axis represents the Shapley additive values for a particular feature displayed on the vertical axis. Positive values indicate that the feature contributes positively to the model prediction for that liver case, while negative values indicate a negative contribution.

Figure 4, Figure 5, Figure 6 and Figure 7 illustrate the developed app and web portal. The mobile app works on any device, whether Android or iOS, and the web portal can be used from a browser or a desktop application. 

### 3.1. Mobile Application

Figure 4 and Figure 5 show some screenshots of the mobile application. After logging in, users can create a new case or access existing ones in their file system. When creating a new case, users take a picture of the liver and enter the donor’s data. Users can also include the percentage of HS estimated by the surgeon (from visual inspection) and the HS estimation according to the biopsy. Among the donor’s data that can be specified, there is the subject’s demographics: sex, age, and body mass index; if steatosis has been confirmed by ultrasound; and biochemical variables: aspartate aminotransferase (AST), alanine aminotransferase (ALT), gamma-glutamyl transferase (GGT), and bilirubin. Users can also specify the cause of death, the need for adrenaline, and the number of days that the donor was in the intensive care unit. When users select “Compute Analysis”, the app runs the HS classification algorithm (Section 2.3). Users can include the donor’s demographic and biochemical variables for the prediction (Figure 4b). 

### 3.2. Image Processing and Data Analysis Tools

From the 362 liver photographs, 7240 patches were extracted and analysed as described in Section 2.3. We obtained the best results in terms of area under the curve (AUC) when combining the image-based features (i.e., colour and texture) with the donor’s data using the random forest classifier and the HS threshold at 15%, achieving an AUC = 0.82 (95% CI: [0.74, 0.89]), with 85% of accuracy (95% CI: [80%, 90%]), 92% precision (95% CI: [87%, 95%]), and 89% recall (95% CI: [85%, 93%]), as seen in Figure 6. The clinical variables that had the greatest impact on the model’s predictions, ranked by mean decrease in impurity, were ALT, BMI, AST, and GGT. The impact of the donor data on the prediction of the model using SHAP values is shown in Figure 7. For the SVM classifier, the accuracy was 76% (95% CI: [70%, 82%]), with 79% for precision (95% CI: [76%, 82%]), and 94% for recall (95% CI: [87%, 97%])). For both the random forest and SVM classifiers, AUC decreased up to 6% when not using clinical donor data. When setting an HS threshold at 30%, the number of non-valid livers significantly decreases (from 53 to 14), which hinders performance (AUC was near 0.65 despite larger accuracy than with threshold 15%). Without gamma correction and colour calibration, classification performance decreased to an accuracy of 83% (95% CI: [78%, 88%]) and an AUC of 0.79 (95% CI: [0.71, 0.87]) on the original images, and dropped to 81% (95% CI: [76%, 87%]) and an AUC of 0.74 (95% CI: [0.66, 0.82]) on the distorted images. We also obtained performance results when working in the RGB colour space instead of the L*a*b* space. However, performance was lower and not reported here.

Surgeons’ assessment of HS achieved lower performance than LiverColor. For HS threshold at 15%, on average, their accuracy was of 73% (95% CI: [65%, 81%]), with 51% precision (CI: [40%, 62%]), and 68% recall (CI: [55%, 81%]), which underscores the significant improvement in classification accuracy achieved by LiverColor in comparison to the surgeons (McNemar’s test with *p*-value < 0.001). For HS threshold at 30%, surgeons’ accuracy was 86% (CI: [80%, 92%]) but with 29% precision CI: [20%, 38%]), and 64% recall (CI: [55%, 73%]).

The computational cost of the classification is less than 3 s on a standard laptop computer and less than 4 s on a state-of-the-art smartphone.

### 3.3. Web Interface

**Figure 8:**
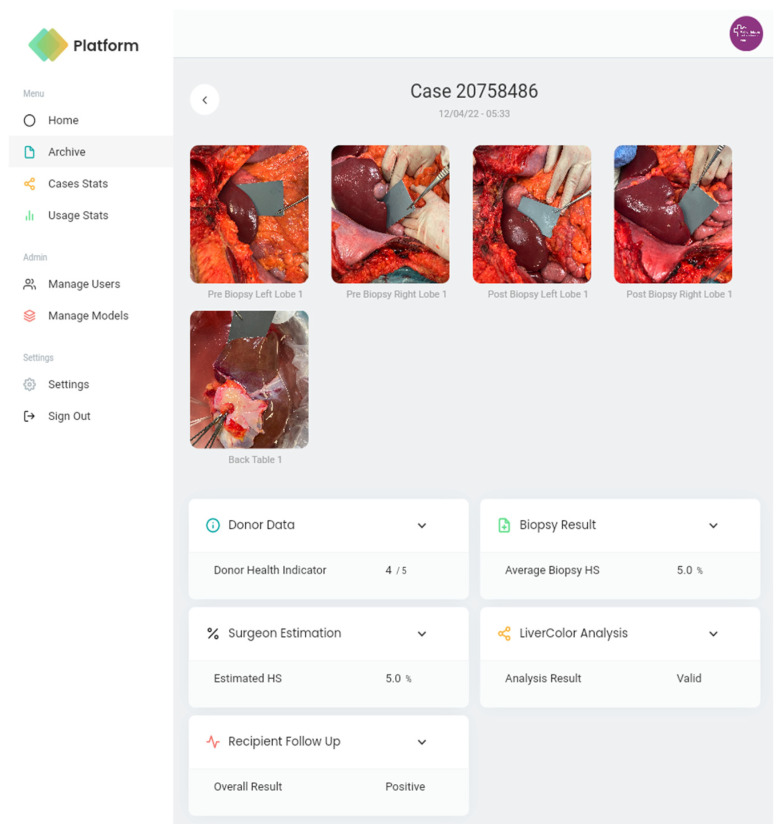
Web portal interface when selecting to visualise a specific case.

**Figure 9:**
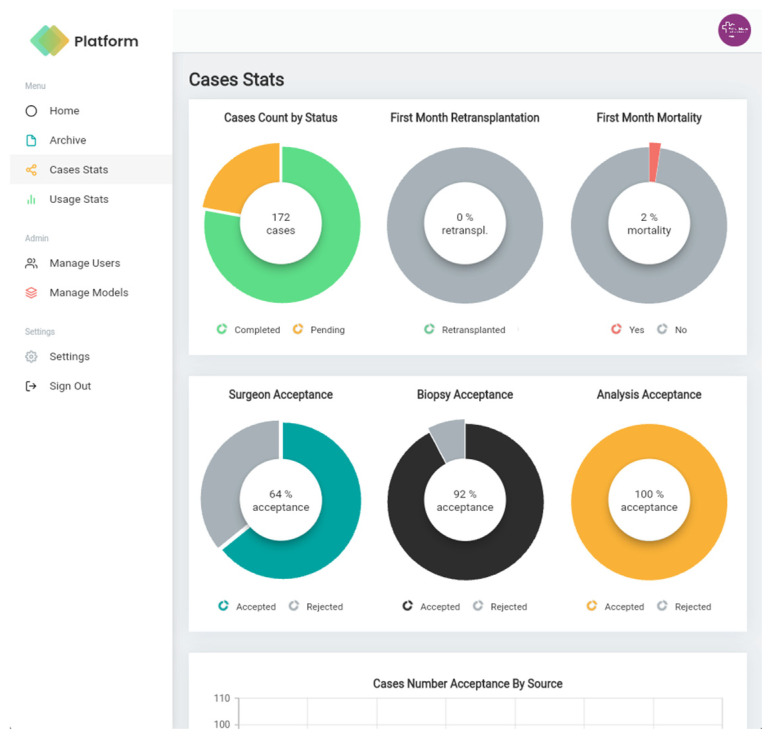
Web portal interface when visualising some statistics of the cases.

Figure 8 and Figure 9 show some screenshots of the web portal interface. The web portal empowers administrators with the ability to track user activity, visualise and analyse registered cases, register new machine learning models, and access key performance indicators for the models in active use.

### 3.4. User Experience

Study participants provided responses to all the statements on the MAUQ questionnaire. Scores on all items were high, ranging from 5 to 7 (maximum), with an average scoring of 6.6. Four out of the nine participants filled the “Additional comments” section, where they valued not only the functionalities related to the assessment of HS, but also the dashboarding to summarise and display procurement-related data. Three participants commented that such a dedicated tool could be particularly beneficial for less experienced surgeons, with two of them highlighting its potential to reduce surgeon’s stress. One participant indicated that not having to use the grey colour card would be an asset to the app.

## 4. Discussion

Our preliminary results show that LiverColor’s performance in predicting HS > 15% is significantly higher than that of the surgeon’s (85% vs. 73% in accuracy, 92% vs. 29% in precision, 89% vs. 64% in recall). 

Comparing with the literature, Adam et al. [2] found that even highly specialised liver procurement surgeons had an accuracy rate of no more than 70% in severe steatosis cases, with positive predictive values of 71%, 46%, and 17% for severe, moderate, and mild steatosis, respectively. Additionally, Yersiz et al. [10] demonstrated that liver transplant surgeons often struggle with accurate classification of moderate and severe steatosis (22.2% and 0%, respectively, in their study of 201 cases). In our study, out of the 26 livers discarded by the surgeons due to their estimate of HS > 30%, 17 (65.3%) were incorrectly discarded, as biopsy results confirmed that HS was actually less than 30%. Furthermore, among the 192 cases, surgeons failed to identify five cases wherein histology indicated that HS > 30%. In contrast, on average, LiverColor overestimated HS in nine cases and underestimated it in five. The results suggest that such a technology could increase donor liver utilisation (i.e., reduce organ waste) and, consequently, facilitate access to liver transplantation, thereby reducing waiting list mortality. 

Although some studies have used liver photographs to assess HS [18,19], to the best of our knowledge, we are the first to present a clinical decision support platform for liver graft assessment. Our platform, implemented both as a cloud-based and a stand-alone application, includes four interrelated components: (1) the database and repository of images and clinical data; (2) the mobile application; (3) the image processing and data analysis tools based on machine learning; and (4) the web portal application. For the image processing pipeline, and in contrast to [18,19], LiverColor includes colour calibration and gamma correction, which improves robustness to lighting conditions. Moreover, LiverColor uses colour features in the L*a*b* colour space, where colours are separated into distinct axes, thereby enabling more precise differentiation of the yellow hues characteristic of steatotic livers. 

Since there are no publicly available databases of liver photographs, benchmarking our classifier tool poses a significant challenge. LiverColor’s accuracy in predicting HS > 30% is 90% (vs 89% in [18] and 99.6% in [19]) with an AUC of 65%, but our low number of cases with HS > 30% (*n* = 14) makes these results less reliable. On the other hand, LiverColor’s accuracy in predicting HS > 15%, a more challenging task according to surgeons, is 85% with an AUC of 0.82. Cesaretti et al. [18] used an SVM classifier to predict HS > 30% from texture features and donor data in a balanced cohort of 54 cases. They achieved an accuracy of 89% but do not provide a ROC analysis or AUC. On the other hand, Ugail et al. [19] used deep learning to extract features followed by an SVM classifier to predict HS > 30% in a nearly balanced cohort of 879 cases. They achieved an accuracy of 99.6% with AUC = 0.99. However, they split the data into training and test sets at the feature level rather than at the donor level, which suggests a potential data leakage causing overfitting. 

LiverColor performs best when using both imaging and clinical data features to predict HS. According to the mean decrease in node impurity in the random forest classifier, the most influential variables are ALT, BMI, AST, and GGT. These variables also ranked highly when computing the SHAP values. Clinical studies have demonstrated their utility as biomarkers in the diagnosis of HS [28,29]. 

From the classification results, we conclude that LiverColor provides a non-invasive, real-time, and accurate assessment of HS in an organ procurement setting. Moreover, it is a flexible tool, allowing for easy incorporation of new descriptors and offering the potential to include assessments of other organs, such as the kidney. 

The development of LiverColor followed a participatory design approach, where the potential users of the app took part in all phases of the design. In particular, LiverColor was extensively validated by several transplant surgeons. They highly appreciated the features provided by the platform, not only its HS assessment functionality but also the dashboarding to summarise and display procurement-related data. Results obtained from the usability test support the launch of upcoming releases of this application to be implemented in real scenarios. Furthermore, clinicians indicated the future positive impact that such a tool may have in the decision-making of organ acceptance, reducing the stress of the transplant team and optimising their work. Since HS is often overestimated by surgeons, our platform could improve the liver donor pool utilisation by avoiding the unnecessary discard of viable organs, thus reducing waiting lists and saving lives.

Thus, although the implemented functionalities are still under improvement and further clinical validation is required, we believe that this model can serve as a steppingstone to develop tools that can be incorporated into the clinical routine of organ procurement. Its integration into mobile and web platforms enhances accessibility, establishing it as a versatile tool for clinicians across various healthcare infrastructures. Its application spans from pre-operative assessments in transplant centres to intra-operative support, delivering real-time, objective evaluations for surgeons. Furthermore, it can function as a triage tool in emergency settings, offering crucial rapid assessments of liver viability. Moreover, in the era of ex-situ organ machine perfusion, it is crucial to identify which grafts will benefit from this procedure to justify its high cost [30]. 

The generalisability of the findings from this study is potentially limited due to several factors. Firstly, the study’s small sample size, especially the low number of livers with HS greater than 30%, and the absence of livers with HS over 60% (severe HS), may not accurately represent the broader population of liver grafts. These limitations can affect the robustness of the results, making it difficult to confidently apply the findings to all clinical scenarios involving liver transplants. Secondly, the study was conducted as a single-centre investigation, which may introduce biases related to the specific practices, protocols, and patient demographics of the institution. Validation on external datasets from multiple centres is crucial to determine whether the model performs consistently across different clinical environments and with diverse patient populations. Further, although the platform demonstrated good accuracy and AUC in the current study, the true performance can only be confirmed through larger-scale studies. These studies should encompass a wider range of liver conditions and be conducted in varied geographical and institutional settings. Such comprehensive validation is essential to ensure the platform’s effectiveness and reliability in real-world clinical applications, thereby enhancing its clinical utility and broadening its adoption in liver transplant assessments. Lastly, another limitation is that we only considered macroestatosis. Microsteatosis is considered to have less negative impact for the outcome of liver transplantation, but some studies suggest that it could be interesting to quantify both types [31,32].

In conclusion, our study shows that colour and texture image analysis coupled with machine learning can help to safely identify valid livers during procurement. We developed LiverColor, a modular co-designed platform that provides a reliable, fast, easy, and cost-effective way to assess the donor’s liver for transplantation. 

## Figures and Tables

**Table 1:** Donor data. Statistics are expressed in median values (Q1–Q3) for continuous variables and absolute numbers (percent) for categorical variables. AST: Aspartate AminoTransferase; ALT: Alanine Transaminase; GGT: Gamma-Glutamyl Transferase.

Donors’ Characteristics	*n* = 192
Age (years)	62 (50.25–71.75)
Male	120 (62.5)
Body mass index (kg^2^/m)	27.1 (24.01–30.3)
AST (IU/L)	37.00 (21.00–66.00)
ALT (IU/L)	26.00 (16.00–57.50)
GGT (IU/L)	42.00 (22.00–82.75)
Bilirubin (mg/dL)	0.5 (0.30–0.71)
Cause of death	
Stroke	78 (45.35)
Head trauma	23 (13.37)
Anoxia	40 (23.26)
Other	31 (18.02)
Discarded for liver transplantation	53 (27.6)
Due to steatosis >30% by surgeons’ point of view	26 (13.54)
Due to other causes	27 (14.06)
Steatosis > 30% by histology	14 (7.3)
Steatosis > 15% by histology	53 (27.6)

## Data Availability

Embargo on data due to commercial restrictions.

## References

[1] Hałoń A., Patrzałek D., Rabczyński J. (2006). Hepatic steatosis in liver transplant donors: Rare phenomenon or common feature of donor population?. Transplant. Proc..

[2] Adam R., Reynes M., Johann M., Morino M., Astarcioglu I., Kafetzis I., Castaing D., Bismuth H. (1991). The outcome of steatotic grafts in liver transplantation. Transplant. Proc..

[3] Feng S. (2008). Steatotic livers for liver transplantation—Life-saving but at a cost. Nat. Rev. Gastroenterol. Hepatol..

[4] Mikolasevic I., Milic S., Filipec-Kanizaj T. (2017). Fatty liver allografts are associated with primary graft non-function and high mortality after transplantation. Liver Int..

[5] Lozanovski V.J., Khajeh E., Fonouni H., Pfeiffenberger J., von Haken R., Brenner T., Mieth M., Schirmacher P., Michalski C.W., Weiss K.H. (2018). The impact of major extended donor criteria on graft failure and patient mortality after liver transplantation. Langenbecks Arch. Surg..

[6] Gedallovich S.M., Ladner D.P., VanWagner L.B. (2022). Liver transplantation in the era of non-alcoholic fatty liver disease/metabolic (dysfunction) associated fatty liver disease: The dilemma of the steatotic liver graft on transplantation and recipient survival. Hepatobiliary Surg. Nutr..

[7] Cotter T.G., Rinella M. (2020). Nonalcoholic Fatty Liver Disease 2020: The State of the Disease. Gastroenterology.

[8] Jadhav P.V., Kothakota S.R., Sasidharan M., Kareem H., Nair A.K. (2020). Effect of Donor Hepatic Steatosis on Ischemia Reperfusion Injury in Liver Transplant Recipient. J. Clin. Exp. Hepatol..

[9] Ratziu V., Charlotte F., Heurtier A., Gombert S., Giral P., Bruckert E., Grimaldi A., Capron F., Poynard T. (2005). LIDO Study Group. Sampling variability of liver biopsy in nonalcoholic fatty liver disease. Gastroenterology.

[10] Yersiz H., Lee C., Kaldas F.M., Hong J.C., Rana A., Schnickel G.T., Wertheim J.A., Zarrinpar A., Agopian V.G., Gornbein J. (2013). Assessment of hepatic steatosis by transplant surgeon and expert pathologist: A prospective, double-blind evaluation of 201 donor livers. Liver Transpl..

[11] Tien C., Remulla D., Kwon Y., Emamaullee J. (2021). Contemporary strategies to assess and manage liver donor steatosis: A review. Curr. Opin. Organ. Transplant..

[12] Imber C.J., St Peter S.D., Lopez I., Guiver L., Friend P.J. (2002). Current practice regarding the use of fatty livers: A trans-Atlantic survey. Liver Transpl..

[13] Rey J.W., Wirges U., Dienes H.P., Fries J.W. (2009). Hepatic steatosis in organ donors: Disparity between surgery and histology?. Transplant. Proc..

[14] Golse N., Cosse N., Allard M., Laurenzi A., Tedeschi M., Guglielmo N., Fernandez-Sevilla E., Robert M., Tréchot B., Pietrasz D. (2019). Evaluation of a micro-spectrometer for the real-time assessment of liver graft with mild-to-moderate macrosteatosis: A proof of concept study. J. Hepatol..

[15] Zhao Q., Lan Y., Yin X., Wang K. (2023). Image-based AI diagnostic performance for fatty liver: A systematic review and meta-analysis. BMC Med. Imaging.

[16] Rajamani A.S., Rammohan A., Sai V.V.R., Rela M. (2022). Current techniques and future trends in the diagnosis of hepatic steatosis in liver donors: A review. Liver Transpl..

[17] Cherchi V., Mea V.D., Terrosu G., Brollo P.P., Pravisani R., Calandra S., Scarpa E., Ventin M., D’Alì L., Lorenzin D. (2022). Assessment of hepatic steatosis based on needle biopsy images from deceased donor livers. Clin. Transplant..

[18] Cesaretti M., Brustia R., Goumard C., Cauchy F., Poté N., Dondero F., Paugam-Burtz C., Durand F., Paradis V., Diaspro A. (2020). Use of artificial intelligence as an innovative method for liver graft macrosteatosis assessment. Liver Transpl..

[19] Ugail H., Abubakar A., Elmahmudi A., Wilson C., Thomson B. (2022). The use of pre-trained deep learning models for the photographic assessment of donor livers for transplantation. Art. Int. Surg..

[20] Beck K., Andres C. (2005). eXtreme Programming explained. Embrace Change.

[21] Ebner M. (2007). Color Constancy.

[22] Isensee F., Jaeger P.F., Kohl S.A., Petersen J., Maier-Hein K.H. (2021). nnU-Net: A self-configuring method for deep learning-based biomedical image segmentation. Nat. Methods.

[23] Meslouhi O., Kardouchi M., Allali H., Gadi T., Benkaddour Y. (2011). Automatic detection and inpainting of specular reflections for colposcopic images. Open Comput. Sci..

[24] Ojala T., Pietikäinen M., Harwood D. (1996). A Comparative Study of Texture Measures with Classification Based on Feature Distributions. Pattern Recognit..

[25] Nielsen J., Molich R. Heuristic evaluation of user interfaces. Proceedings of the ACM of Human Factors in Computing Systems.

[26] Alwashmi M.F., Hawboldt J., Davis E., Fetters M.D. (2019). The iterative convergent design for mobile health usability testing: Mixed methods approach. JMIR Mhealth Uhealth.

[27] Zhou L., Bao J., Setiawan I.M.A., Saptono A., Parmanto B. (2019). The mHealth App Usability Questionnaire (MAUQ): Development and Validation Study. JMIR Mhealth Uhealth.

[28] Noureddin M., Loomba R. (2012). Nonalcoholic fatty liver disease: Indications for liver biopsy and noninvasive biomarkers. Clin. Liver Dis..

[29] Wilkins T., Tadkod A., Hepburn I., Schade R.R. (2013). Nonalcoholic fatty liver disease: Diagnosis and management. Am. Fam. Physician.

[30] Webb A., Lester E., Shapiro A.M.J., Eurich D., Bigam D.L. (2022). Cost-utility analysis of normothermic machine perfusion compared to static cold storage in liver transplantation in the Canadian setting. Am. J. Transplant..

[31] Nishida S., Misawa R., Okumura K., Sogawa H., Bodin R., Veillette G., Matsukawa M., Lansman S. (2023). Steatosis assessment in donor livers. Transplantation.

[32] Croome K.P., Lee D.D., Croome S., Nakhleh R.E., Abader Sedki Senada P., Livingston D., Yataco M., Taner C.B. (2019). Does Donor Allograft Microsteatosis Matter? Comparison of Outcomes in Liver Transplantation with a Propensity-Matched Cohort. Liver Transpl..

